# Dr. L. S. Parmly

**Published:** 1859-10

**Authors:** 


*Obituary.*—It is with sincere sorrow that we announce the death of Dr. L. S. Parmly, late of New Orleans, and brother of Dr. Eleazar Parmly, of New York. Intelligence of this sad event reached us just as our last sheets were preparing for the press. Dr. Parmly commenced his professional career about forty-five years ago, and for a long time enjoyed a large and lucrative practice in New Orleans; but, impressed with the belief that prevention was better than cure, he devoted the best energies of his active mind to the study of hygienic dentistry, availing himself of every opportunity to impress upon the minds of his patients the importance of observing such rules as he had found to be most conducive to the preservation of the health of the teeth and gums. To this department of his calling the last fifteen or eighteen years of his life were chiefly devoted, and that too, at pecuniary sacrifices, which few, even of the most philanthropic, would be willing to make.

During the last five or six years, Dr. P. spent most of his time in Paris, and although stricken down with paralysis about a year and a half ago, he recovered sufficiently to see and converse with many who sought the benefit of his professional advice. At what time he died, or whether in Paris or New York, we have not yet learned.

Dr. Parmly wrote several small treatises on the teeth. The first, entitled "Guide to the Management of the Teeth," &c., was published in 1819. The second, entitled "Lectures on the Natural History and Management of the Teeth, the Causes of their Decay," &c., was published in 1821. Besides the above, he wrote several tracts on the best means of securing the preservation of the health of the teeth.

It was the writer's good fortune to be acquainted with Dr. P. for nearly thirty years, and never did he know a man more philanthropically devoted to his calling, or possessed of more excellent qualities of heart.

We hope to be able, in our next No , to give a more extended account of the life of our departed friend.

